# Role of Baicalin and Liver X Receptor Alpha in the Formation of Cholesterol Gallstones in Mice

**DOI:** 10.1155/2020/1343969

**Published:** 2020-04-21

**Authors:** Geng Chen, Shuodong Wu

**Affiliations:** ^1^Department of General Surgery, Shengjing Hospital of China Medical University, Shenyang 110004, China; ^2^Department of General Surgery, The First Affiliated Hospital of Dalian Medical University, Dalian 116600, China

## Abstract

This study was aimed at investigating the effect of baicalin on experimental cholesterol gallstones in mice. The mouse gallstone model was induced by feeding with a lithogenic diet, and cholesterol stones were found in the gallbladder. The lithogenic diet caused elevation of triglycerides, cholesterol, and low-density lipoprotein concentrations and descent of high-density lipoprotein concentration in serum. Hyperplasia and inflammatory infiltration were observed in the gallbladder wall of lithogenic diet-fed mice. We also found the increase of cholesterol content and the decrease of bile acid in bile. Real-time PCR and western blot results demonstrated that the expression levels of two enzymes (cholesterol 7*α*-hydroxylase (CYP7a1) and sterol 12*α*-hydroxylase (CYP8b1)) to catalyze the synthesis of bile acid from cholesterol were decreased and that two cholesterol transporters (ATP-binding cassette transporter G5/G8 (ABCG5/8)) were increased in the liver of lithogenic diet-fed mice. The lithogenic diet also led to enhanced activity of alanine aminotransferase and aspartate aminotransferase in serum; increased concentrations of tumor necrosis factor-*α*, interleukin- (IL-) 1*β*, IL-6, and malondialdehyde; and decreased superoxide dismutase activity in the liver, suggesting inflammatory and oxidative stress. In addition, liver X receptor alpha (LXR*α*) was increased in the liver. After gavage of baicalin, the lithogenic diet-induced gallstones, hyperlipidemia, gallbladder hyperplasia, inflammation, and oxidative stress in liver and cholesterol metabolism disorders were all alleviated to some degree. The expression of LXR*α* in the liver was inhibited by baicalin. In addition, the LXR*α* agonist T0901317 aggravated lithogenic diet-induced harmful symptoms in mice, including the increase of gallstone formation, hyperlipidemia, hepatic injury, inflammation, and oxidative stress. In conclusion, we demonstrated that baicalin played a protective role in a lithogenic diet-induced gallstone mouse model, which may be mediated by inhibition of LXR*α* activity. These findings may provide novel insights for prevention and therapy of gallstones in the clinic.

## 1. Introduction

The gallstone (cholelithiasis) is a common digestive disease, affecting 10-20% of the global adult population [1]. The gallstones are classified based on composition and location. More than 90% of gallstones are gallbladder cholesterol stones [1]. Bile is essential for food digestion, containing bile salts, phospholipids, cholesterol, proteins, and bilirubin. Bile is an aqueous colloidal system, and phospholipids and cholesterol are presented in the bile as mixed micelles [2]. Bile is produced in the liver and is secreted into the duodenum to digest food. During the interdigestive interval, the bile is stored and concentrated in the gallbladder. Alterations in the proportions of components lead to phase separation of cholesterol from the solution in bile. Under suitable physicochemical conditions, the excess phase-separated cholesterol can aggregate to form lamellar liquid crystals, and eventually, cholesterol monohydrate crystals are separated out. These crystals form cholesterol gallstones by agglomeration within a gallbladder-secreted mucin gel [2, 3].

At present, the primary treatments for symptomatic gallbladder cholesterol stones are cholecystectomy, extracorporeal shockwave lithotripsy, and medical dissolution [4]. Accumulating evidences have shown that some medicines can regulate the cholesterol level and alleviate experimental gallstone formation in rodents, such as the *Lysimachia christinae* aqueous extract and schaftoside [5, 6]. Baicalin is a monomeric flavonoid compound, isolated from *Scutellaria baicalensis georgi*. Previously, *Scutellaria baicalensis georgi* extracts were found to exert a protective effect on the liver [7, 8]. Notably, baicalin has been demonstrated with anti-inflammatory, antiapoptotic, and antioxidative roles [9–11]. It has been reported that baicalin could alleviate high fat diet-induced hyperlipidemia and liver dysfunction in mice [12]. In a high fat diet-induced rabbit atherosclerosis model, baicalin attenuated the lipid accumulation and formation of atherosclerotic plaques in carotid arteries and promoted the expression of cholesterol transporters and cholesterol export in macrophages [13]. Because of the roles in the regulation of cholesterol metabolism, baicalin was hypothesized to function in gallstone formation.

Liver X receptors (LXRs) are nuclear receptors that are critical for the control of lipid homeostasis. LXRs serve as cholesterol sensors that regulate the expression of multiple genes involved in the efflux, transport, and excretion of cholesterol [14]. LXRs can be activated by physiological concentrations of sterol metabolites and bind to their target DNA sequences to regulate their transcription [14]. It has been reported that activation of LXRs promotes biliary cholesterol secretion by upregulating the hepatic ATP-binding cassette transporter G5 (ABCG5) and ABCG8, which are transports of cholesterol [15]. Cholesterol 7*α*-hydroxylase (CYP7a1), an enzyme that catalyzes the rate-limiting step of bile acid synthesis from cholesterol, is also a target of LXRs. LXR*α* (-/-) mice fail to induce transcription of *cyp7a1* [16]. LXR*α* and LXR*β* share considerable sequence homology and respond to the same endogenous ligands. LXR*α* is highly expressed in the liver, adipose tissue, and macrophages, and LXR*β* is expressed in all tissues examined. Therefore, we detected that the expression of LXR*α* and its targets that are involved in cholesterol metabolism in the liver of mice received a lithogenic diet and investigated its roles in cholesterol gallstone formation.

In the current study, to investigate the roles of baicalin and LXR*α* in the formation of cholesterol gallstones, the mouse gallstone model was established. The effects of baicalin and LXR*α* on gallstone formation, hyperlipidemia, hepatic injury, inflammation, and cholesterol metabolism were evaluated in mice. We hypothesize that baicalin may be useful for the prevention of cholesterol gallstones, which may be mediated by LXR*α* inhibition in mice.

## 2. Material and Methods

### 2.1. Ethical Statement

The animals in this study were purchased from Huafukang Biotechnology Co., Ltd. (license no.: SCXK (Beijing, China) 2014-0004) and taken care of according to the Guide for the Care and Use of Laboratory Animals (8th Edition, NIH). The experimental procedure was approved by the Ethics Committee of Shengjing Hospital of China Medical University (registration number: 2017PS231K).

### 2.2. Animals

**Figure 1:**
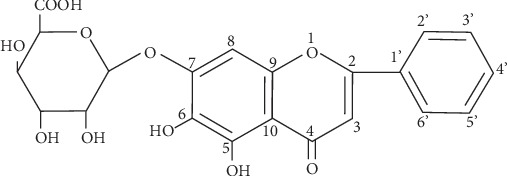
The structure of baicalin.

Healthy C57BL/6 mice of 6 weeks old were kept in a controlled environment (22-24°C with 12 : 12 h light/dark cycles) with free access to food and water. After acclimatization for one week, the mice were randomly divided into five groups: control, 100 mg/kg baicalin (BA(H)), gallstone, gallstone+50 mg/kg baicalin (BA(L)), and gallstone+BA(H). For the mouse gallstone model, the gallstone formation was induced as previously described [17–19]. Briefly, the mice in gallstone groups received a self-made lithogenic diet (a standard diet supplemented with 15.8% fat, 1.25% cholesterol, and 0.5% sodium cholate) for 8 weeks, and the control mice were fed with a standard diet (containing 20% protein, 4% fat, 48% carbohydrate, and essential vitamins and microelements) (HUANYU Bio, Beijing, China). The mice in the BA(H) and gallstone+BA(H) groups were administered by oral gavage with baicalin of 100 mg/kg every day and mice in the gallstone+BA(L) group with baicalin of 50 mg/kg. Baicalin was purchased from Aladdin Biochemical Technology Co. Ltd. (cat. no. B11021, Shanghai, China), and its structure is shown in Figure 1. The solution should be prepared when it is to be used, and storage for a long time should be avoided. After 7 days, the body weight of the mice was recorded every 7 days for 8 weeks. Finally, all mice were sacrificed. The vein blood and bile were collected, and the liver and gallbladder were isolated for the subsequent experiments.

For another batch of detection, the mice in the gallstone+T0901317 group were fed with a lithogenic diet and received an oral gavage of LXR*α* agonist T0901317 (20 mg/kg every day) (cat. no.: 293754-55-9, MCE, China) for 8 weeks.

### 2.3. Measurement of Serum Lipid Profiles

The content of triglyceride (TG), total cholesterol (TC), high-density lipoprotein cholesterol (HDL-C), low-density lipoprotein cholesterol (LDL-C), and total bile acid (TBA) in the serum was detected with commercially available kits (Jiancheng, Nanjing, Jiangsu, China) according to the manufacturer's protocols.

The activities of aspartate aminotransferase (AST) and alanine aminotransferase (ALT) were determinated using AST and ALT assay kits (Jiancheng, Nanjing, Jiangsu, China). The standard curve of enzymatic activity was drawn according to the manufacturer's instruction. After dilution, the serum sample (5 *μ*L) was incubated with a substrate buffer (20 *μ*L) at 37°C for 30 min, reacted with 2,4-dinitrophenylhydrazine buffer (20 *μ*L) at 37°C for 20 min, and finally mixed with 0.4 mol/L NaOH (200 *μ*L) at room temperature for 15 min. The optical density (OD) of the solution was measured at 510 nm. The AST and ALT activities were calculated based on the OD510.

### 2.4. Analysis of Plasma Concentration of Inflammatory Markers

The total protein concentration was detected using a BCA protein assay kit (cat. no. P0011, Beyotime, Haimen, Jiangsu, China). The standard curve of tumor necrosis factor- (TNF-) *α* was drawn with a TNF-*α* standard substance of graded concentrations. The sample was incubated with TNF-*α* antibody at room temperature for 90 min. After washing, the sample was incubated with HRP-labeled streptavidin for 30 min and reacted with a TMB substrate in the dark for 20 min until the addition of a stop buffer. The OD450 and OD570 of the solution were determined, and the content of TNF-*α* was calculated according to the standard curve by an ELISA kit (cat. no. EK282/3, Multisciences, Hangzhou, Zhejiang, China). The standard curve of interleukin- (IL-) 1*β* was drawn with the IL-1*β* standard substance of graded concentrations, and the IL-1*β* content in the sample was determined with an ELISA kit (cat. no. EK201B/3, Multisciences, Hangzhou, Zhejiang, China) as previously described. The IL-6 content in the sample was detected using an ELISA kit (cat. no. EK206/3, Multisciences, Hangzhou, Zhejiang, China) according to the previous description.

### 2.5. Determination of Superoxide Dismutase (SOD) Activity and Malondialdehyde (MDA) Content

The enzymatic activity of SOD and MDA content was detected using a total SOD assay kit (cat. no. A001-1, Jiancheng, Nanjing, Jiangsu, China) and MDA assay kit (cat. no. A003-1, Jiancheng Nanjing, Jiangsu, China) according to the manufacturer's instructions, respectively.

### 2.6. Gene Expression Analysis

The total RNA was extracted with a TriPure reagent kit (cat. no. RP1001, BioTeke, Beijing, China), and the concentration and purity were identified with the NanoDrop 2000 ultraviolet spectrophotometer (Thermo Scientific, Waltham, MA, USA). Subsequently, the RNA was reversely transcribed into cDNA using a M-MLV reverse transcriptase (cat. no. PR6502, BioTeke, Beijing, China), in the presence of Oligo (dT) and random primers. The cDNA was used for real-time PCR with 2x Power Taq PCR MasterMix (cat. no. PR1702, BioTeke, Beijing, China) and SYBR Green (cat. no. S9430, Sigma, St. Louis, MO, USA), to detect the mRNA levels of CYP7a1, sterol 12*α*-hydroxylase (CYP8b1), and LXR*α*, with *β*-actin as the internal control. The PCR procedure was set as follows: 94°C for 5 min 15 s, 60°C for 25 s, and 72°C for 30 s, followed with 40 cycles of 72°C for 5 min 30 s and 40°C for 2 min 30 s, melting at 60-94°C for 1 s each 1°C, and finally incubated at 25°C for several minutes. The sequence information of PCR primers is shown in Table 1.

### 2.7. Protein Abundance Analysis

The cellular protein was extracted with the western blot and IP cell lysis buffer with 1 mM PMSF (Beyotime, Haimen, Jiangsu, China). The protein sample was separated with SDS-PAGE and transferred onto a PVDF membrane (cat. no. IPVH00010, Millipore, Boston, MA, USA). After blocking with skim milk at room temperature for 1 h, the membrane was incubated with one of the antibodies at 4°C overnight: rabbit anti-CYP7a1 (1 : 500; cat. no. A10615, ABclonal, Wuhan, Hubei, China), rabbit anti-CYP8b1 (1 : 1000; cat. no. ab191910, Abcam, Cambridge, UK), rabbit anti-ABCG5 (1 : 500; cat. no. A8589, ABclonal, Wuhan, Hubei, China), rabbit anti-ABCG8 (1 : 500; cat. no. A1880, ABclonal, Wuhan, Hubei, China), rabbit anti-LXR*α* (1 : 1000; cat. no. ab41902, Abcam, Cambridge, UK), and mouse anti-*β*-actin (1 : 1000; cat. no. sc-47778, Santa Cruz, CA, USA). After rinsing with TBST, the membrane was incubated with goat anti-rabbit or anti-mouse secondary antibody labeled with HRP (1 : 5000; cat. no. A0208 or A0216, Beyotime, Haimen, Jiangsu, China) at 37°C for 45 min. Subsequently, the membrane was reacted with ECL reagent (cat. no. P0018, Beyotime, Haimen, Jiangsu, China) for 5 min and followed with a signal exposure in the dark. The bands were analyzed with Gel-Pro Analyzer software. *β*-Actin served as the internal control.

### 2.8. Immunohistochemistry Staining

The liver tissue was fixed with formaldehyde overnight and washed with flow water for 4 h. The tissue was dehydrated with graded concentrations of ethanol, permeated with xylene, embedded with paraffin, and cut into sections of 5 *μ*m. The sections were dewaxed with xylene and ethanol and reacted with antigen repair buffer in boiling for 10 min. After blocking with 3% H_2_O_2_ for 15 min and goat serum for 15 min, the sections were incubated with rabbit antibody against LXR*α* (1 : 200; cat. no. ab41902, Abcam, Cambridge, UK) at 4°C overnight. Then, the sections were incubated with goat secondary antibody labeled HRP at 37°C for 60 min and reacted with DAB reagent (cat. no. DA1010, Solarbio, Beijing, China) for several minutes. After counterstaining with hematoxylin (cat. no. H8070, Solarbio, Beijing, China) for 3 min, the sections were soaked in 1% hydrochloric acid/ethanol for 3 s and dehydrated with ethanol and xylene. Finally, the sections were mounted with gum and observed with a microscope (cat. no. BX53, Olympus, Tokyo, Japan) at 400x magnification.

### 2.9. Hematoxylin-Eosin (HE) Staining

The gallbladder or liver tissue was made into paraffin sections as previously described. The sections were dewaxed with xylene and ethanol and stained with hematoxylin for 5 min. Then, the sections were soaked in 1% hydrochloric acid/ethanol for 3 s and stained with eosin (cat. no. A600190, Sangon, Shanghai, China) for 3 min. After dehydrating with ethanol and xylene, the sections were mounted with gum and observed with a microscope (cat. no. BX53, Olympus, Tokyo, Japan) at 200x magnification.

## 3. Statistical Analysis

The data in this study were presented as mean ± SD, with six individuals. The data were analyzed by one-way ANOVA test, with post hoc Bonferroni's multiple comparisons. A *p* value less than 0.05 was considered statistically significant (^∗^*p* < 0.05, ^∗∗^*p* < 0.01, ^∗∗∗^*p* < 0.001, and ns: no significance).

## 4. Results

### 4.1. Lithogenic Diet Induced Stone Formation in Gallbladder and Hyperlipidemia in Mice

**Figure 2:**
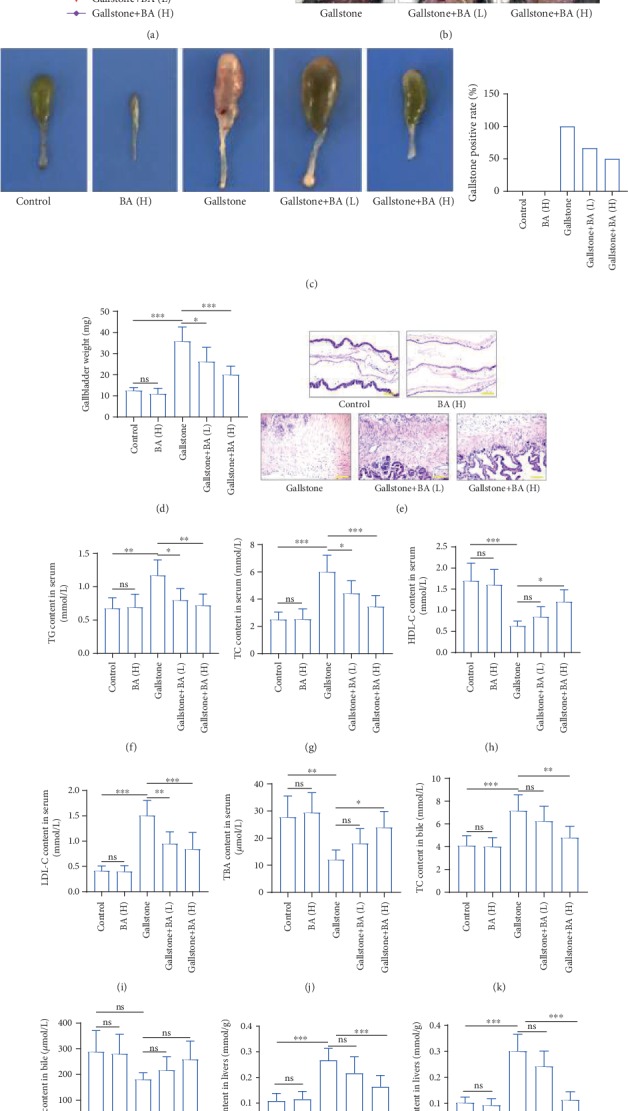
Baicalin alleviated lithogenic diet-induced gallstone and hyperlipidemia in mice. (a) Body weight was recorded and calculated in the mice, which received a lithogenic diet or/and Baicalin treatment. *n* = 6. (b, c) The representative pictures of the gallbladder of mice with a lithogenic diet or/and Baicalin administration (the arrows indicate the gallbladder). The gallstone positive rate of mice is shown in each group. *n* = 12. (d) The weight of gallbladders was calculated. (e) HE staining was performed to detect the pathological and physiological characteristics of gallbladder tissues (the scale bar represented 100 *μ*m). (f–j) The content of triglyceride (TG) (f), total cholesterol (TC) (g), high-density lipoprotein cholesterol (HDL-C) (h), low-density lipoprotein cholesterol (LDL-C) (i), and total bile acid (TBA) in serum (j) of mice in each group. (k, l) The content of TC (k) and TBA (l) in bile of mice in each group. *n* = 6. (m, n) The content of total cholesterol (TC) (m) and triglyceride (TG) (n) in the liver of mice in each group. *n* = 6 (^∗^*p* < 0.05, ^∗∗^*p* < 0.01, and ^∗∗∗^*p* < 0.001). ns: no significance; BA(L): 50 mg/kg baicalin; BA(H): 100 mg/kg baicalin.

To induce gallstone formation, the mice were fed with lithogenic diet for 8 weeks. The body weight of mice was recorded, and the results showed that lithogenic diet induced the increase of body weight, whereas baicalin treatment alleviated the body weight in mice (Figure 2(a)). Figures 2(b) and 2(c) showed the gallbladder with different treatments. Lithogenic diet led to 100% stone formation in the gallbladder, which was significantly reduced by baicalin gavage (Figure 2(c)). Consistently, the gallbladder weight was obviously increased in mice after receiving lithogenic diet, which was decreased by baicalin administration (Figure 2(d)). HE staining revealed that lithogenic diet caused hyperplasia and inflammatory infiltration in gallbladder wall tissues, which was alleviated by baicalin administration (Figure 2(e)). Subsequently, the data showed that the levels of TG, TC, and LDL-C in serum were significantly increased, while the HDL-C and TBA levels were decreased in lithogenic diet-fed mice, which suggested hyperlipemia. The changes of serum lipid levels were reversed by administration of baicalin (Figures 2(f)–2(j)). Notably, the baicalin treatment alone hardly affects the characteristics of the gallbladder and serum lipid levels. In addition, the cholesterol content in bile and the liver was increased, and bile acid content in bile was decreased in lithogenic diet-fed mice (Figures 2(k)–2(n)), suggesting cholesterol metabolism disorders.

### 4.2. Baicalin Regulated Cholesterol Metabolism in Liver

**Figure 3:**
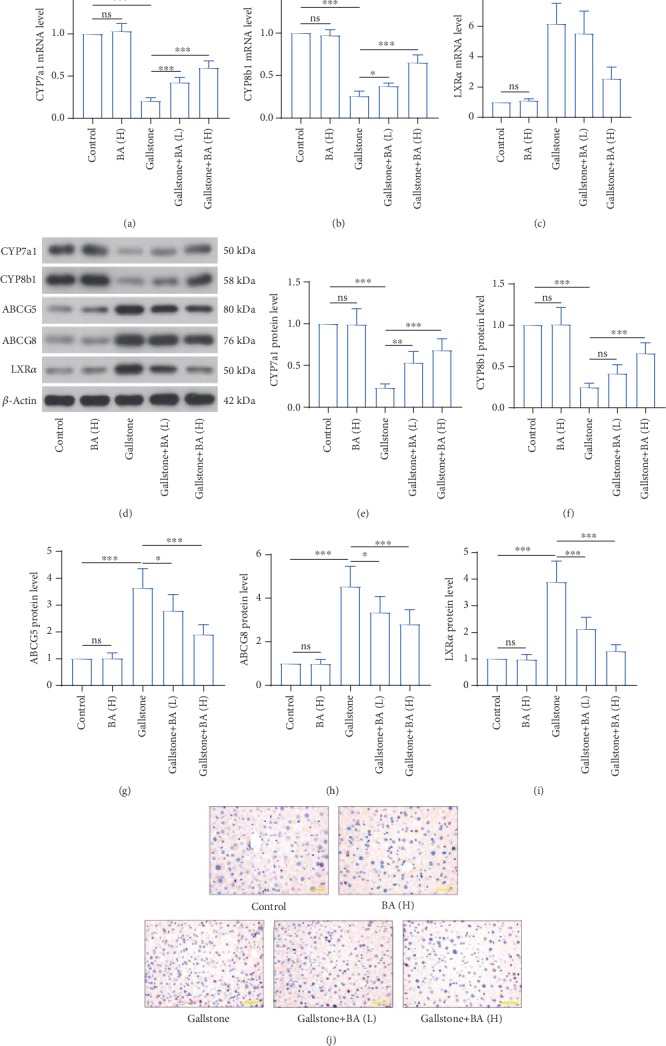
Lithogenic diet induced disorders of cholesterol synthesis and transport in liver. (a–c) The mRNA levels of cholesterol 7*α*-hydroxylase (CYP7a1) (a), sterol 12*α*-hydroxylase (CYP8b1) (b), and liver X receptor *α* (LXR*α*) (c) in the liver were detected by real-time PCR. (d) Western blot was used to measure the protein levels of CYP7a1, CYP8b1, adenosine triphosphate binding cassette (ABC)G5, ABCG8, and LXR*α* in the liver of mice that received a lithogenic diet or Baicalin administration. (e–i) Quantitative analysis of CYP7a1 (e), CYP8b1 (f), ABCG5 (g), ABCG8 (h), and LXR*α* (i) of immunoblotting bands in (d). (j) Immunohistochemistry staining was used for detection of LXR*α* expression in the liver (the scale bar represented 50 *μ*m). *n* = 6 (^∗^*p* < 0.05, ^∗∗^*p* < 0.01, and ^∗∗∗^*p* < 0.001). ns: no significance; BA(L): 50 mg/kg baicalin; BA(H): 100 mg/kg baicalin.

We then detected the expression levels of several proteins involving in cholesterol transport and synthesis of bile acid from cholesterol. The real-time PCR and western blot results revealed that the mRNA and protein levels of CYP7a1 and CYP8b1 were decreased more than 75% in the liver of mice that received the lithogenic diet, compared with the control (Figures 3(a), 3(b), and 3(d)–3(f)), which suggested reduced synthesis from cholesterol to bile acid. The expression levels of two cholesterol transporters, ABCG5 and ABCG8, were increased over 3-fold in the liver of lithogenic diet-fed mice, compared to the control (Figures 3(d), 3(g), and 3(h)). We also detected a critical protein regulating cholesterol metabolism, LXR*α*. The results showed that the transcriptional and translational levels of LXR*α* in the liver were increased 6-fold and 3.9-fold after the lithogenic diet (Figures 3(c), 3(d), and 3(i)). The immunohistochemistry staining also confirmed the expression change of LXR*α* in the liver (Figure 3(j)). The changes of CYP7a1, CYP8b1, ABCG5, ABCG8, and LXR*α* were all reversed by baicalin administration to some degree.

### 4.3. Baicalin Attenuated Lithogenic Diet-Induced Hepatic Damage

**Figure 4:**
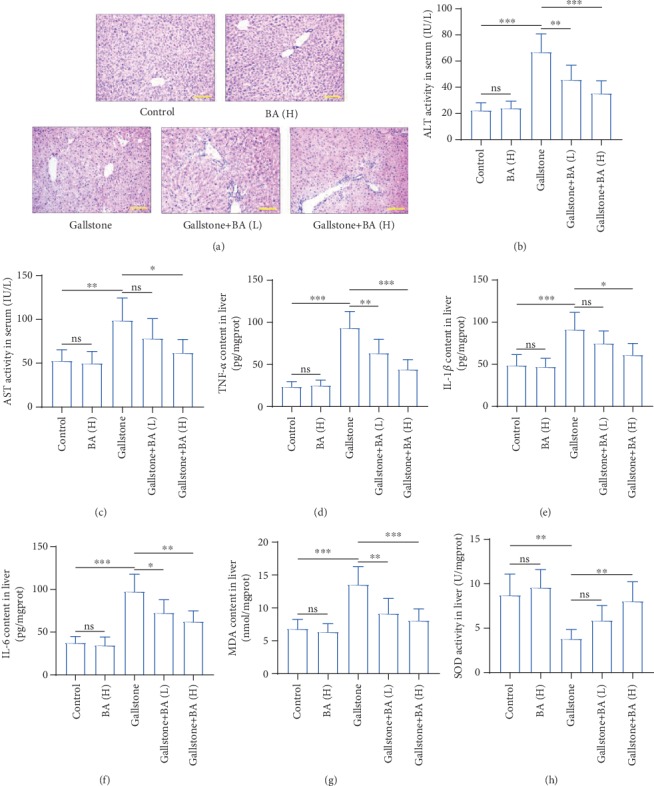
Baicalin attenuated lithogenic diet-induced damage in liver. (a) HE staining was performed to determine the pathological and physiological characteristics of the liver (the scale bar represented 100 *μ*m). (b, c) The activity of alanine aminotransferase (ALT) (b) and aspartate aminotransferase (AST) (c) in serum. (d–f) The content of inflammatory factors tumor necrosis factor- (TNF-) *α* (d), interleukin- (IL-) 1*β* (e), and IL-6 (f) in the liver was detected by ELISA. (g) The malondialdehyde (MDA) content in the liver. (h) The enzymatic activity of superoxide dismutase (SOD) in the liver. *n* = 6 (^∗^*p* < 0.05, ^∗∗^*p* < 0.01, and ^∗∗∗^*p* < 0.001). ns: no significance; BA(L): 50 mg/kg baicalin; BA(H): 100 mg/kg baicalin.

We also examined the hepatic damage in lithogenic diet-fed mice. HE staining showed that the lithogenic diet induced cell necrosis and inflammatory infiltration, which was restrained by baicalin gavage (Figure 4(a)). The enzymatic activity of ALT and AST, two markers of hepatic damage, was increased approximately 3-fold and 2-fold in the serum, respectively (Figures 4(b) and 4(c)). Several inflammatory factors were determined by ELISA. The data revealed that the content of TNF-*α*, IL-1*β*, and IL-6 was increased in the liver of lithogenic diet-fed mice (Figures 4(d)–4(f)). In addition, the MDA content was increased and SOD activity was decreased, suggesting oxidative stress in the liver (Figures 4(g) and 4(h)). The lithogenic diet-induced injury, inflammation, and oxidative stress in the liver were all ameliorated by baicalin application to some degree.

### 4.4. Role of Baicalin and LXR*α* in the Formation of Cholesterol Gallstones in Mice

**Figure 5:**
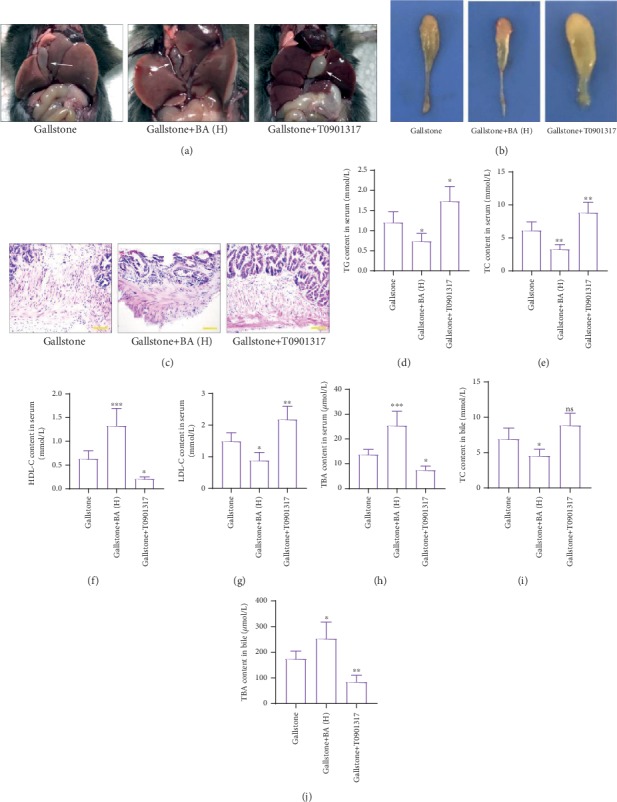
Baicalin restrained gallstone formation by inhibiting LXR*α*. (a, b) The representative pictures of gallbladders in mice with lithogenic diet and baicalin or LXR*α* agonist T0901317 (the arrows indicate the gallbladder). (c) HE staining was performed to detect the pathological and physiological characteristics of the gallbladders of mice in each group (the scale bar represented 100 *μ*m). (d–i) The content of TG (d), TC (e), HDL-C (f), LDL-C (g), and TBA (h) in serum. (i, j) The content of TC (i) and TBA (j) in bile. *n* = 6 (^∗^*p* < 0.05, ^∗∗^*p* < 0.01, and ^∗∗∗^*p* < 0.001). ns: no significance; BA(H): 100 mg/kg baicalin.

**Figure 6:**
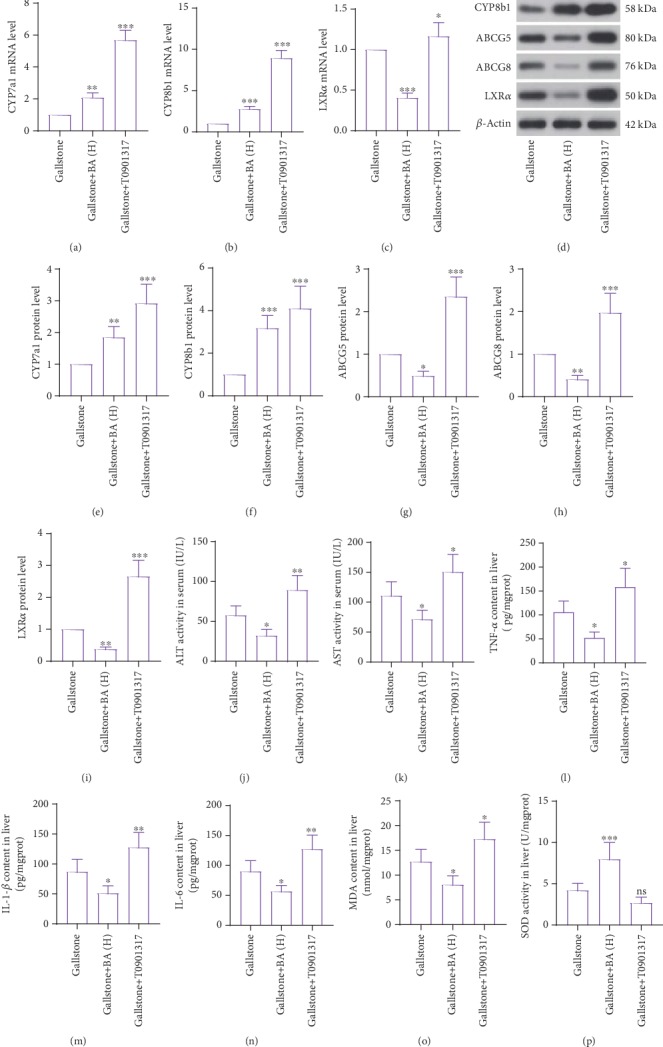
Baicalin regulated cholesterol metabolism and lithogenic diet-induced hepatic damage by inactivating LXR*α*. (a–c) Real-time PCR was used for detection of mRNA levels of CYP7a1 (a), CYP8b1 (b), and LXR*α* (c) in the liver of mice with a lithogenic diet and baicalin or LXR*α* agonist T0900307. (d) The protein levels of CYP7a1, CYP8b1, ABCG5, ABCG8, and LXR*α* were measured by western blot. (e–i) Quantitative analysis of CYP7a1 (e), CYP8b1 (f), ABCG5 (g), ABCG8 (h), and LXR*α* (i) of immunoblotting bands in (d). (j, k) The enzymatic activity of ALT (j) and AST (k) in serum. (l–n) The content of inflammatory factors TNF-*α*, IL-1*β*, and IL-6 in the liver was detected by ELISA. (o) The MDA content in the liver. *n* = 6. (p) The SOD activity in the liver. ^∗^*p* < 0.05, ^∗∗^*p* < 0.01, and ^∗∗∗^*p* < 0.001. ns: no significance; BA(H): 100 mg/kg baicalin.

Next, we tried to investigate how baicalin played its roles in lithogenic diet-fed mice. Because of the important roles of LXR*α* in cholesterol metabolism, an LXR*α* agonist T0901317 was used as a positive control. As shown in Figures 5(a) and 5(b), the T0901317 administration aggravated the lithogenic diet-induced gallstone formation and gallbladder wall hyperplasia, which were contrary to baicalin's effects. The increased serum TG, TC, and LDL-C levels in lithogenic diet-fed mice were reduced by baicalin, but further elevated by T0901317 (Figures 5(c), 5(d), and 5(f)). The decreased serum HDL-C and TBA levels in lithogenic diet-fed mice were raised by baicalin, but further reduced by T0901317 (Figures 5(g) and 5(h)). T0901317 also promoted the TC content and reduced the bile acid content in bile (Figures 5(i) and 5(j)). Real-time PCR results showed that T0901317 promoted the transcription of CYP7a1, CYP8b1, and LXR*α* in the liver of lithogenic diet-fed mice (Figures 6(a)–6(c)). Western blot results revealed that T0901317 enhanced translation of CYP7a1, CYP8b1, ABCG5, ABCG8, and LXR*α* (Figures 6(d)–6(i)). The detection of ALT and AST activity demonstrated that T0901317 aggravated hepatic injury in lithogenic diet-fed mice (Figures 6(j) and 6(k)). ELISA detection results of TNF-*α*, IL-1*β*, and IL-6 revealed that T0901317 accelerated lithogenic diet-induced inflammation in the liver (Figures 6(l)–6(n)). The MDA content and SOD activity in the liver demonstrated that the oxidative stress in the gallbladder was further promoted by T0901317 administration (Figures 6(o) and 6(p)). The results in this section suggested that the effects of T0901317 on gallstone formation, hyperlipidemia, hepatic injury, inflammation, and cholesterol metabolism were opposite to baicalin.

## 5. Discussion

Here, we demonstrated that the lithogenic diet induced the increased levels of TG, cholesterol, and LDL-C and a decreased level of HDL-C in serum, suggesting hyperlipemia and hypercholesteremia in mice. Moreover, baicalin regulated cholesterol metabolism and attenuated lithogenic diet-induced hepatic damage and inflammation, whereas the LXR*α* agonist T0901317 exhibited opposite effects in mice.

The cholesterol synthesized in the liver can be excreted with transporters. ABC transporters are divided into importers and exporters, involved in nutrient and ion intake and metabolite and signaling molecular exporting [20]. Among the ABCA-ABCG transporters, several transporters are responsible for the transport of cholesterol. ABCG5/G8 heterodimers promote efficient secretion of cholesterol and plant sterols from hepatocytes into bile [21]. It has been reported that overexpression of ABCG5 and ABCG8 elevated biliary cholesterol levels by more than 5-fold in mice [22], and extremely low biliary cholesterol concentrations were observed in mice lacking ABCG5 or ABCG8 [23]. The expression of ABCG5 and ABCG8 is enhanced by LXRs [24]. Moreover, in response to dietary cholesterol, LXR*α* was identified to be required for the elevation of mouse ABCG5 and ABCG8 expression [25]. In our study, a lithogenic diet led to increased expression of ABCG5 and ABCG8 in the liver, accompanied with elevated cholesterol content in bile and stone formation in the gallbladder. After administration of the LXR*α* agonist, the ABCG5 and ABCG8 expression levels were further increased, and the gallstone formation was also promoted. Although some LXR targets in mice are not conserved in humans, these results revealed that LXR*α* promoted cholesterol transport from the liver to bile and gallstone formation by upregulating ABCG5 and ABCG8 expression levels, which might provide a new direction in the treatment of gallstones.

Cholesterol could be synthesized into bile acid in the liver. CYP7a1 is the rate-limiting enzyme in the classic bile acid biosynthesis pathway, which catalyzes the hydroxylation at 7*α*-position of cholesterol. Next, microsomal 3*β*-hydroxy-C27-steroid dehydrogenase/isomerase (3*β*-HSD) converts 7*α*-hydroxycholesterol to 7*α*-hydroxy-4-cholestene-3-one, which is converted to 7*α*,12*α*-dihydroxy-4-cholesten-3-one by CYP8b1 [26]. CYP7a1 and CYP8b1 are regulated by many nuclear receptors, including LXR, farnesoid X receptor (FXR), *α*-fetoprotein transcription factor (FTF), pregnane X receptor (PXR), and peroxisome proliferator activated receptor (PPAR) [26, 27]. The transcription of *cyp7a1* and *cyp8b1* genes is also regulated by cholesterol and bile acid levels [27]. In our study, the lithogenic diet caused decreased expression of CYP7a1 and CYP8b1, which suggested inhibited synthesis of bile acid. As expected, the bile acid content in serum and bile was decreased. After LXR*α* agonist administration, the expression levels of CYP7a1 and CYP8b1 were increased, but the bile acid content in serum and bile was decreased. We speculated that the LXR*α* enhanced both cholesterol excretion and bile acid synthesis, but the rate of bile acid elevation was not as fast as that of cholesterol elevation. CYP7a1 is the first target of LXR*α*, and its expression is positively regulated by LXR*α*. However, their expression levels in lithogenic diet-fed mice were not consistent, as well as in other reports [15, 28]. This contradiction may be due to the addition of bile acid. CYP7a1 was found to initiate the main bile acid biosynthetic (classic or neutral) pathway expressed in the liver, whereas CYP27a1 participated in the alternative (or acidic) pathway expressed in many tissues [26]. Thus, to investigate the role of baicalin on the formation of cholesterol gallstones by bile acid synthesis pathways, further experiments are needed to verify this speculation in the future.

The baicalin gavage in lithogenic diet-fed mice promoted the expression of CYP7a1 and CYP8b1 and reduced that of ABCG5 and ABCG8 in the liver, which resulted in increased bile acid synthesis and decreased cholesterol secretion, evidenced by the elevation of bile acid content and decline of cholesterol content in bile. Moreover, baicalin also decreased the expression level of LXR*α*, and the LXR*α* agonist promoted gallstone formation and hyperlipidemia. So we speculated that baicalin functioned by inhibiting LXR*α* activity. In a high fat-induced rabbit atherosclerosis model, baicalin enhanced the expression of LXR*α* and inhibited the formation of atherosclerotic plaques in carotid arteries [13]. These suggested that the effect of baicalin on LXR*α* may depend on disease types. It has been reported that both the LXR*α* transgene and the LXR*α* agonist aggravated lithogenic diet-induced gallstone formation and hyperlipidemia [15]. Baicalin has been reported to regulate lipid metabolism and ameliorated non-alcoholic steatohepatitis [29], atherosclerosis [13], and high fat diet-induced obesity [12]. In this study, baicalin alleviated lithogenic diet-induced stone formation in the gallbladder and hyperlipidemia and regulated cholesterol metabolism, and baicalin may play its roles by inhibiting LXR*α* activity. However, due to the concomitant treatment of baicalin and the LXR*α* agonist in a lithogenic diet-induced gallstone mouse model, more experiments will be performed to further clarify the mechanism of LXR inhibition underlying the beneficial effect of baicalin in the future.

Dietary cholesterol is absorbed in intestine, and the cholesterol transporters ABCG5/8 are also expressed in enterocytes in the proximal small intestine. The expression levels of ABCG5 and ABCG8 were increased both in the liver and in the intestine in lithogenic diet-fed mice [25]. It will be helpful for our study to detect the expression of ABCG5/8 in the intestine and the cholesterol component in feces. These examinations will be performed in the near future in a separate study.

In addition, the formation of gallstone is often accompanied by damage of the liver and gallbladder. In our study, the lithogenic diet led to hyperplasia in the gallbladder wall and inflammation and oxidative stress in the liver, which were attenuated by baicalin administration. The treatment of LXR*α* agonist aggravated the damage in the liver and gallbladder, consistent with the previous results.

In summary, our results demonstrated that baicalin alleviated lithogenic diet-induced gallstone information, hyperlipidemia, and damage in the gallbladder and liver and regulated cholesterol metabolism. The roles of LXR*α* agonist T0901317 administration were contrary to baicalin's effects. The data suggest that baicalin caused LXR*α* inhibition and thereby protected against the formation of cholesterol gallstones in mice.

## Figures and Tables

**Table 1:** The sequence information of primers used in this study.

Name	Sequence
CYP7a1	Forward: 5′-AAGACCGCACATAAAGCC-3′
	Reverse: 5′-GATGCCCAGAGGATCACG-3′

CYP8b1	Forward: 5′-ACCTGTTTCTGGGTCCTC-3′
	Reverse: 5′-TCTCCTCCATCACGCTGT-3′

LXR*α*	Forward: 5′-AGCGGCAAGAAGAGGAAC-3′
	Reverse: 5′-CAGGCGGTCTGAGAAGGA-3′

*β*-Actin	Forward: 5′-AATCGTGCGTGACATCAA-3′
	Reverse: 5′-AGAAGGAAGGCTGGAAAA-3′

## Data Availability

The data used to support the findings of this study are available from the corresponding author upon request.
